# Supplementary material for: Complex‐centric proteome profiling by SEC‐SWATH‐MS
Source: Mol Syst Biol. 2019 Jan 14;15(1):e8438. doi: 10.15252/msb.20188438 (PMC6346213; doi:10.15252/msb.20188438)
Supplement: Supplementary file 7 — Dataset EV6 [file MSB-15-e8438-s007.zip › feature_plots_bioplex/P57775.pdf]

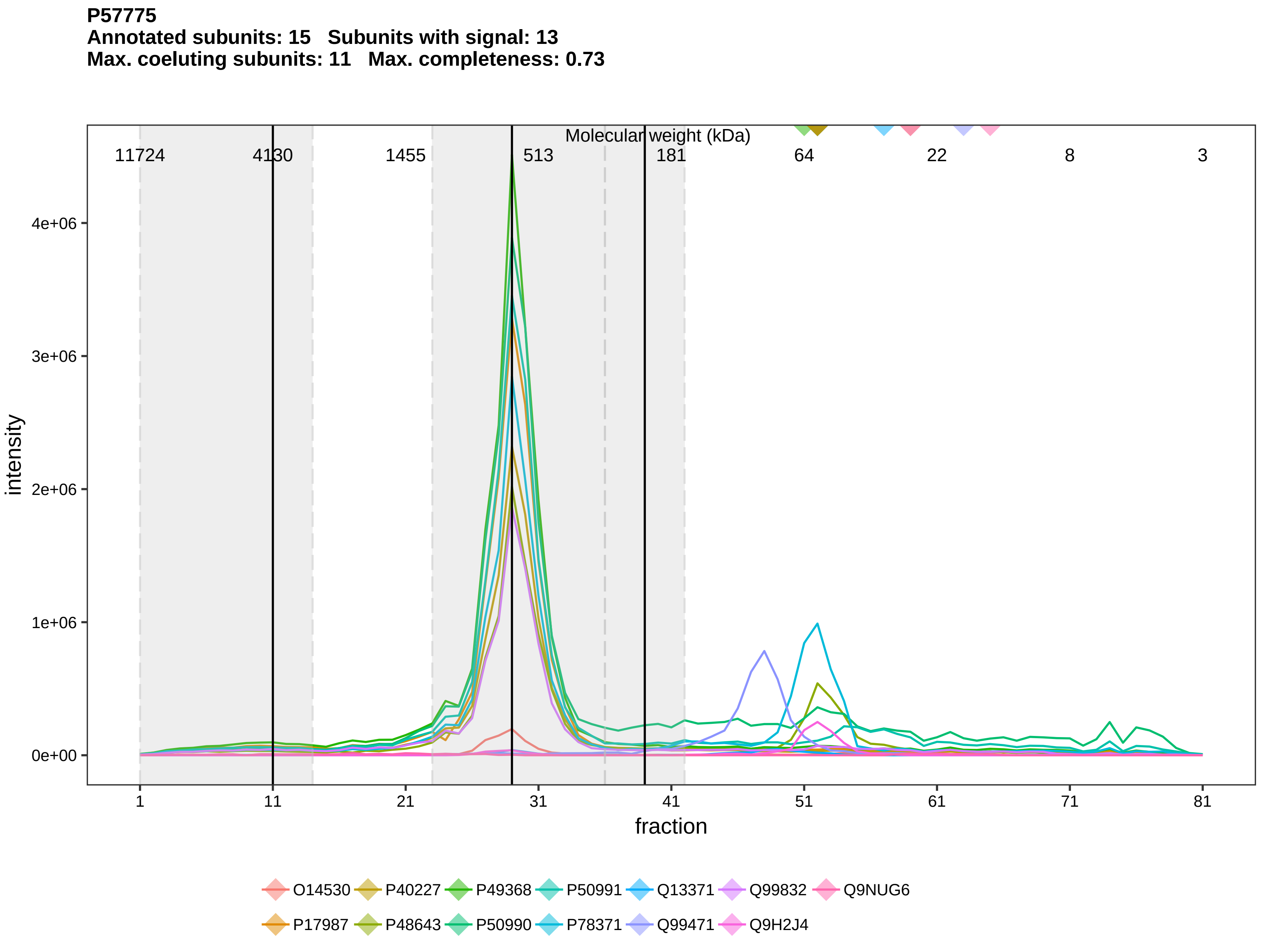

P57775
Annotated subunits: 15 Subunits with signal: 13
Max. coeluting subunits: 11 Max. completeness: 0.73
Molecular weight (kDa)
11724
4130
1455
513
181
64
22
8
3
intensity
4e+06
3e+06
2e+06
1e+06
0e+00
1
11
21
31
41
51
61
71
81
fraction
O14530
P40227
P49368
P50991
Q13371
Q99832
Q9NUG6
P17987
P48643
P50990
P78371
Q99471
Q9H2J4
